# Low-Cost Arduino Reverse Transcriptase Loop-Mediated Isothermal Amplification (RT-LAMP) for Sensitive Nucleic Acid Detection

**DOI:** 10.3390/bios14030128

**Published:** 2024-02-29

**Authors:** Bruno Dias Camargo, Mateus Cassaboni Stracke, Heloisa Bruna Soligo Sanchuki, Viviane Klassen de Oliveira, Hellen Cristina Ancelmo, Dayanne Mozaner Bordin, Fabricio Klerynton Marchini, Emilson Ribeiro Viana, Lucas Blanes

**Affiliations:** 1 Laboratory for Applied Science and Technology in Health, Carlos Chagas Institute, Oswaldo Cruz Foundation (Fiocruz), Professor Algacyr Munhoz Mader 3775 St., Curitiba 81350-010, Brazil; bruno.camargo@ibmp.org.br (B.D.C.); mateus.stracke@ibmp.org.br (M.C.S.); heloisa.sanchuki@ibmp.org.br (H.B.S.S.); hellen.ancelmo@ibmp.org.br (H.C.A.); fabricio.marchini@ibmp.org.br (F.K.M.); lucas.blanes@fiocruz.br (L.B.); 2 Paraná Institute of Molecular Biology, Professor Algacyr Munhoz Mader 3775 St., Curitiba 81350-010, Brazil; klassen.viviane@gmail.com; 3 School of Mathematical and Physical Sciences, Faculty of Science, University of Technology Sydney, Ultimo, NSW 2007, Australia; 4 Academic Department of Physics (DAFIS), Federal University of Technology—Paraná (UTFPR), Sete de Setembro 3165 Av., Curitiba 80230-901, Brazil; emilsonjunior@utfpr.edu.br

**Keywords:** Arduino, RT-LAMP, reverse transcriptase loop-mediated isothermal amplification, nucleic acid detection, isothermal amplification

## Abstract

This work presents a low-cost transcription loop-mediated isothermal amplification (RT-LAMP) instrument for nucleic acid detection, employing an Arduino Nano microcontroller. The cooling system includes customized printed circuit boards (PCBs) that serve as electrical resistors and incorporate fans. An aluminum block is designed to accommodate eight vials. The system also includes two PCB heaters—one for sample heating and the other for vial lid heating to prevent condensation. The color detection system comprises a TCS3200 color 8-sensor array coupled to one side of the aluminum heater body and a white 8-LED array coupled to the other side, controlled by two Multiplexer/Demultiplexer devices. LED light passes through the sample, reaching the color sensor and conveying color information crucial for detection. The top board is maintained at 110 ± 2 °C, while the bottom board is held at 65 ± 0.5 °C throughout the RT-LAMP assay. Validation tests successfully demonstrated the efficacy of the colorimetric RT-LAMP reactions using SARS-CoV-2 RNA amplification as a sample viability test, achieving 100% sensitivity and 97.3% specificity with 66 clinical samples. Our instrument offers a cost-effective (USD 100) solution with automated result interpretation and superior sensitivity compared to visual inspection. While the prototype was tested with SARS-CoV-2 RNA samples, its versatility extends to detecting other pathogens using alternative primers, showcasing its potential for broader applications in biosensing.

## 1. Introduction

Polymerase chain reaction (PCR) is the gold standard for RNA detection methods. This technique is well-known for its specificity and reliance on trained personnel [1]. In the pursuit of a new generation of PCR, the ultrafast photonics PCR method reduces thermocycling time by employing various nanomaterials such as AuNPs, Au nanofilms, and Fe_3_O_4_ nanoagglomerates [2]. However, those methods present limitations, including elevated costs, extended processing times, and the requirement for controlled environments and specialized equipment [3]. To overcome these limitations, point-of-care (POC) tests have emerged as promising alternatives. 

POC instruments and techniques are characterized by their rapid analysis, cost-effectiveness, and high sensitivity. These characteristics position POC tests as alternatives to traditional methodologies. Furthermore, the capacity for seamless integration into resource-limited settings and the ability to generate reliable results with minimal operator training are also crucial characteristics of POC tests [4].

In addition to RT-PCR, another NAAT (nucleic acid amplification techniques) method on the rise is RT-LAMP (reverse transcription loop-mediated isothermal amplification). This represents an economical, simple, and highly sensitive POC technique for RNA detection [5]. The method is based on DNA synthesis with auto cycling chain displacement, leading to exponential amplification. The reaction is facilitated by four to six sets of primers, and the entire process takes place within 30–40 min under isothermal conditions (65 °C) [6]. This technique often combines colorimetric detection methods, employing fluorescent dyes and pH indicators for efficient and cost-effective results [7,8]. Phenol red, a pH indicator included in LAMP kits, plays a vital role in the RT-LAMP color reaction. As the amplification of RNA nucleic acid occurs, the reaction exhibits a noticeable color shift from pink (pH 8.8) to yellow (pH < 6.8), indicating media acidification due to the generation of hydrogen ions. It is important to note that LAMP reactions with phenol red provide a visual indicator of the amplification process; however, the reaction is inherently non-quantitative. This combination is advantageous over traditional methods, especially when used with POC instruments, simplifying the diagnostic process and removing complex equipment requirements [9].

Implementing colorimetric RT-LAMP to achieve quicker and simpler detection highlights the importance of POC equipment having automated result interpretation capabilities. Recent literature highlights the integration of the LAMP methods with POC devices. Wan et al. (2019) developed a POC device featuring LAMP and microfluidic technology for DNA detection [10]. In 2023, Nguyen et al. [11] developed a system specifically designed for SARS-CoV-2 detection. In this context, the utilization of a light-to-frequency color sensor is documented in the literature for various applications such as bacteria color detection [12], cyanide quantification [13], urine testing [14] with fluorescence detection on a lab-on-a-chip device [15], and on a lateral flow immunoassay (LFIA) reader [16].

The critical importance of precise temperature control in PCR processes has been widely recognized in the literature [16,17,18]. To address this requirement, PID (proportional–integral–derivative) algorithms have emerged as the technology of choice for achieving the necessary precision [18]. The PID system operates through a controlled feedback loop mechanism, a well-established approach in various applications, including industrial systems [19] and PCR instruments [20], as well as POC PCR instruments [21]. The PID system incorporates a continuous feedback loop mechanism where a temperature sensor consistently provides input. The electronic device in the system continually computes the error value, representing the disparity between the desired setpoint and the measured temperature. Subsequently, it applies corrections based on the PID equation, as elucidated by Johnson and Moradi (2006) [22]. This control system ensures precise and dynamic temperature regulation, an essential factor in the success of PCR experiments and related applications.

Arduino, an open-source electronics platform, controls electronic circuits by sending data and instructions to a microcontroller, often the ATmega328 [23]. It comprises a programmable circuit board (microcontroller) and associated software running on a computer, enabling the writing and uploading of computer codes onto the physical board [24]. Arduino boards read and respond to inputs and manipulate outputs, and their use is widespread in various projects, including those involving temperature control with PID libraries [25,26,27], as well as color detection using TCS3200.

Current literature provides limited information on cost-effective portable PCR devices that use conventional PCR vials [7,28,29]. In contrast, a large number of high-end commercially available instruments can reach a substantial cost [19].

In this study, we developed a cost-effective RT-LAMP instrument with an approximate cost of USD 100. The instrument incorporates PCBs as heating elements, aluminium blocks capable of holding eight vials, a color detection system, and an Organic Light Emitting Diode (OLED) for results display. It is equipped with a PID temperature control system for enhanced precision. The setup is controlled and monitored by an Arduino microcontroller board. In contrast to commercially available instruments, this low-cost prototype offers a practical and affordable alternative for POC applications, particularly relevant in the context of viral global pandemics such as the COVID-19 pandemic. Proof-of-concept tests were performed using SARS-CoV-2 RNA samples in RT-LAMP reactions and compared with the standard PCR method to evaluate the correlation between the results obtained from our prototype and the standard commercial method.

## 2. Materials and Methods

### 2.1. Samples and Ethical Statement

The SARS-CoV-2 clinical samples were collected from 66 symptomatic patients from Erasto Gaertner Hospital (Curitiba—Brazil) with the Local Ethics Committee approval (CAAE 31592620.4.3001.5248 and 31592620.4.1001.0098). Sample handling and experiments were performed following ANVISA (National Health Surveillance Agency) guidelines and the Brazilian guidelines, and all recruited patients have written a consent statement.

Nasal and oral swabs were collected from each symptomatic patient in 3 mL of phosphate-buffered saline (PBS) in 15 mL centrifuge tubes. Samples were kept at −20 °C until analysis. The RNA extraction was performed with the QIAmp Viral RNA Mini Kit (Qiagen, Hilden, Germany) as described by the manufacturers. The RNA was diluted in ultrapure water to avoid interfering with the colorimetric reaction. The clinical samples were confirmed by RT-qPCR, with Ct values for the E gene and RdRp gene ranging from 13.38 to 33.00 and from 15.12 to 32.86, respectively. All samples had a Ct for internal control RNAse P less than 33 [30].

### 2.2. RT-LAMP Block Device

**Figure 1:**
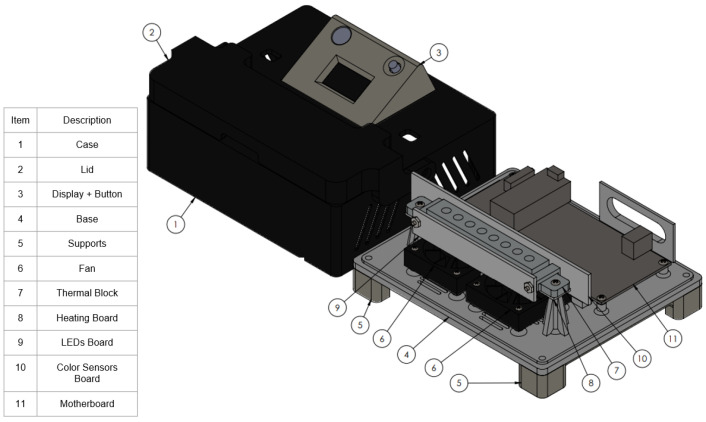
RT-LAMP instrument 3D view with internal components. (1) Case; (2) Lid; (3) Display + Button; (4) Base; (5) Supports; (6) Fan; (7) Thermal Block; (8) Heating Board; (9) LED Board; (10) Color Sensor Board; and (11) Motherboard.

The RT-LAMP equipment, as illustrated in Figure 1, consists of a housing fabricated through additive manufacturing with PLC filament. Internally, it incorporates an RT-LAMP temperature control block, an Arduino Nano microcontroller, and a display, along with color sensors.

### 2.3. Hardware

**Figure 2:**
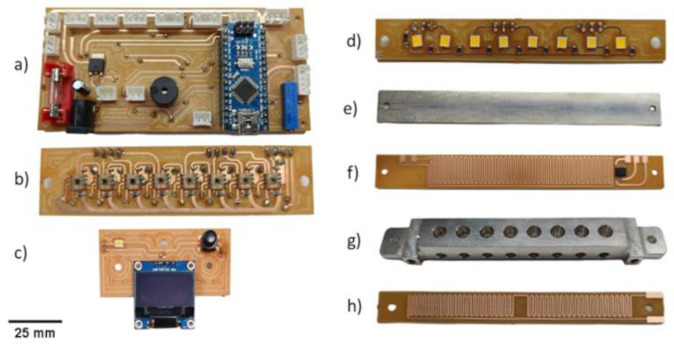
Instrument internal components: (**a**) Mainboard with the Arduino nano microcontroller; (**b**) eight TCS3200 color sensor array boards; (**c**) OLED display, button, and LED indicator boards; (**d**) eight white LED array boards; (**e**) top aluminum; (**f**) top heater board with an LM35 temperature sensor; (**g**) bottom aluminum block; (**h**) bottom heater.

The RT-LAMP block was built using a milled aluminium block (Figure 2g) and designed using the 3D CAD software SolidWorks^®^ 2018. Subsequently, it was coupled with a PCB board with 114.0 × 18.0 × 10.5 mm dimensions. The aluminum block was employed as a heat conductor to ensure uniform heat distribution across the block. Similarly, the top heater featured a thin aluminum plate coupled with a PCB, serving the same purpose of ensuring even heat distribution.

The lowermost aluminium block featured eight evenly distributed vertical conical structures positioned in the central region of its body. Aligned with these conical structures, the block incorporates eight horizontal paths, facilitating the passage of LED light through the microtubes and ultimately reaching the TCS3200 color sensor. An LM35 TO220 temperature sensor was securely fixed to one end of the block. The heating block was fixed to the instrument housing at both ends, while thin horizontal markings were present to secure the emitter and receiver plates for the colorimetric detection process.

Two double-side 35 μm copper FR4 PCB boards were designed and milled using the LPKF S103 (LPKF Laser & Electronics, Suhl, Germany). The electronic designs were created using Software Eagle (Autodesk, Version 9.6.2, Inc., San Rafael, CA, USA) and AutoCAD 2018.1 (Autodesk, Inc., San Rafael, CA, USA). On the end PCB board, a copper resistor with a total resistance of 2.5 Ω was designed, while the top PCB board incorporated a resistor with a resistance of 2.8 Ω. The resistor configuration was arranged in a “zigzag” pattern to enhance heat distribution. A Surface Mount Device (SMD) SOIC8 LM35 temperature sensor was soldered near the resistor on the upper PCB board to improve the accuracy of temperature acquisition.

### 2.4. Electronics

The mainboard was equipped with an Arduino Nano microcontroller to control the entire system. Power modulation to the bottom and top heaters was facilitated by two IRL3103S MOSFETs through PWM signals, while control over the computer fans (coolers) was managed by two BC817 transistors. Each heating system incorporated an LM35 sensor for temperature acquisition, with one sensor featuring a TO220 encapsulation and the other a SOIC-8 encapsulation. The system included eight TCS3200 color sensors utilizing the I2C protocol for communication. These sensors were connected to the mainboard via a 74HC151D Mux/Demux (Multiplexer/Demultiplexer) device, enabling individual reading by one Arduino Nano pin. Similarly, the LED array, comprising eight white SMD LEDs, was connected using a Mux/Demux device, allowing one Arduino Nano pin to illuminate all eight LEDs sequentially. This single pin for both reading and controlling eight components contributed to the efficient utilization of the Arduino Nano board. Figure 2 below illustrates the main components of the developed system.

**Figure 3:**
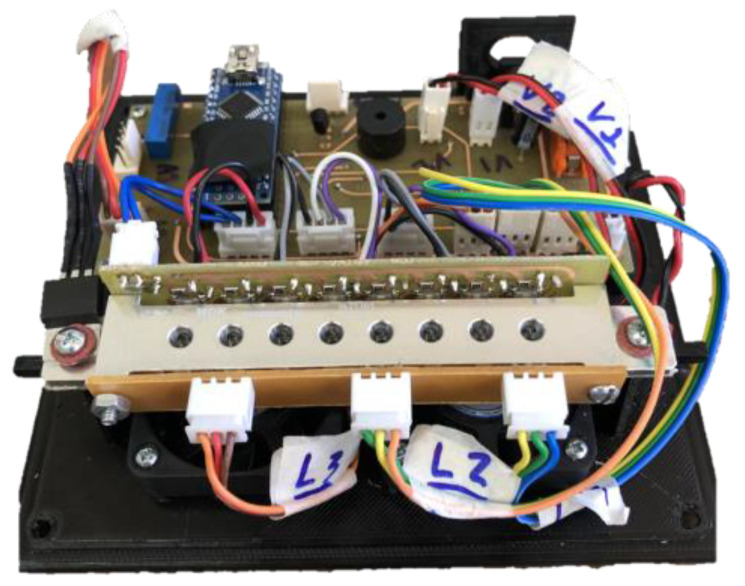
Internal part of the equipment with colorimetric detection system.

The set of components shown in Figure 2, when coupled, forms the colorimetric detection system presented in Figure 3.

**Figure 4:**
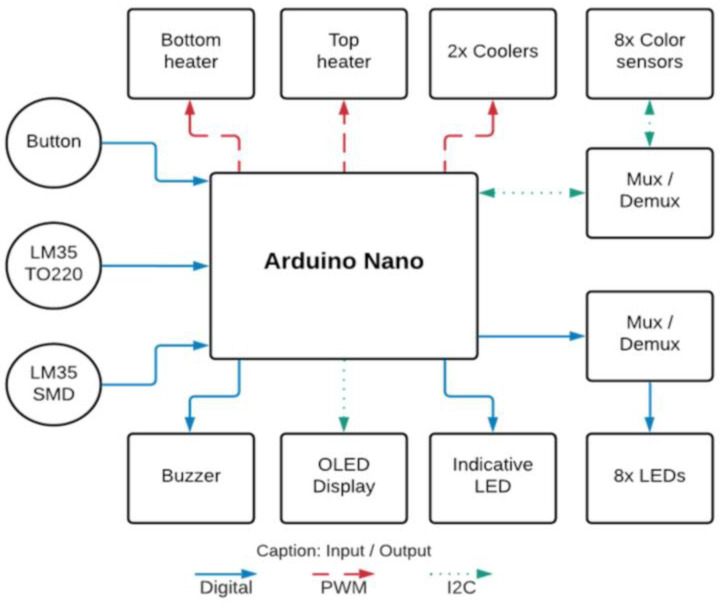
Instrument electronics block diagram.

The instrument was powered by an AC/DC adapter (input: 100–240 V, frequency: 50/60 Hz, output: 12 V, 5 A, FY, W-T5000—manufactured in Shenzhen, China). The block diagram of the instrument electronics is presented in Figure 4, with arrows indicating the direction of signal flow.

### 2.5. Software and Data Processing

The software was developed in C++ for Arduino^®^ Nano, based on I2C communication using the Wire library. For user interaction, an OLED display was implemented using the Adafruit_GFX library to visualize information about the equipment’s step-by-step process, detected temperature, reaction time, and detection results.

The equipment operation control was implemented through a button; a buzzer was incorporated to signalize stage transitions; and an LED was used to indicate the current stage of the equipment.

For temperature control, a proportional integral derivative (PID) controller algorithm was implemented. This algorithm receives the current temperature from the sensor, calculates the parameters, and provides feedback with the resulting signal to the resistor. The logic construction was carried out using the PDI_v1 library.

The board temperature was measured using an LM35 (an analog temperature sensor) coupled to the aluminum block for the calibration process. Simultaneously, the liquid temperature was measured employing a K-type thermocouple connected to an external measurement device (Novus 1200 temperature controller). To simulate real conditions, this external sensor was placed inside a sealed 200 μL PCR Eppendorf tube containing 25 μL of water.

In addition to all those circuits, a few more components are used to clean and stabilize the circuit signals, like filtering capacitors, and a fuse is used for circuit safety.

### 2.6. Colour Detection

A TCS3200 light-to-frequency color sensor operating in its blue and green detection mode was used for color detection. A PCB board was manufactured following the previously described method, but with a distinct design to accommodate the color sensor terminals. The PCB was designed to integrate eight TCS3200 color sensors strategically positioned with uniform distribution, accompanying filter capacitors, and current-limiting resistors. An array board for the TCS3200 operates alongside an LED array board. The LED array board mirrors the design of the TCS3200 board but is tailored specifically to accommodate eight Surface Mount Device (SMD) LEDs, each with its own corresponding resistor.

### 2.7. RT-LAMP Reaction

The primer set used for SARS-CoV-2 detection and the RT-LAMP reaction conditions were previously standardized by our research group [30]. The primer set was first reported by Rabe and Cepko (2020) [31] and was designed for the ORF1a gene (Supplementary Table S1).

Colorimetric RT-LAMP reactions were conducted employing the Colorimetric WarmStart^®^ 2X Master Mix (NEB, Ipswich, USA). The final reaction mixture consisted of FIP (Forward Inner Primer) and BIP (Backward Inner Primer) primers at a final concentration of 1.6 μM each, FOP (Forward Outer Primer) and BOP (Backward Outer Primer) at 0.2 μM each, and FL and BL at 0.4 μM each, along with 5 μL of RNA sample in a total final volume of 25 μL. The incubation of reactions occurred for 30 min at 65 °C using the RT-LAMP instrument and a conventional thermocycler. Subsequently, after the designated reaction period, the samples were cooled to room temperature (25 °C). Positive controls for PCR were employed as positive samples, while a non-template control (NTC) with nuclease-free water was used as the negative control. The clinical RNA was extracted using the QIAamp^®^ RNA viral Mini Kit (Qiagen, Hilden, Germany), following the manufacturer’s instructions.

### 2.8. Housing

**Figure 5:**
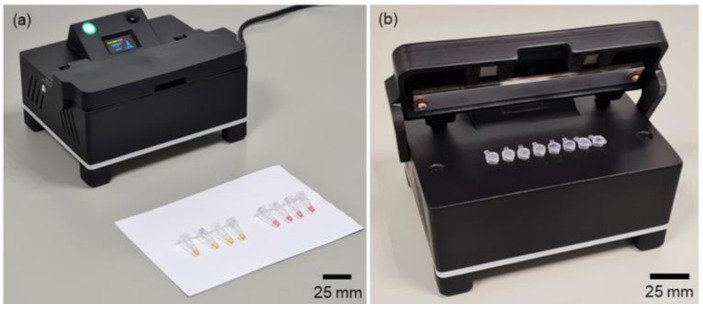
Instrument housing that holds the electronics main board, the LAMP heating/detection block, the lid heater, the fans, and the OLED display. (**a**) Close-lid during tests; (**b**) open-lid to insert/remove the microtubes.

The instrument housing (Figure 5) was designed for user friendliness while accommodating all the previously mentioned components. The electronic mainboard is located at the back part of the instrument, facilitating connection with other peripherals, and the front side hosts the RT-LAMP heating and detection block, with fans strategically placed directly below it.

To ensure optimal airflow during the cooling process and prevent electronic components from overheating, the housing features openings on all sides and an elevated structure.

The top of the instrument contains the OLED display and an indicative LED, and the control button was designed with a 30° angle to enhance the user experience. A lid at the top incorporates a thick layer of printed material to shield users from potential burns due to elevated temperatures. The lid has two pairs of neodymium magnets to secure it when closed.

## 3. Results and Discussion

### 3.1. Temperature Control

**Figure 6:**
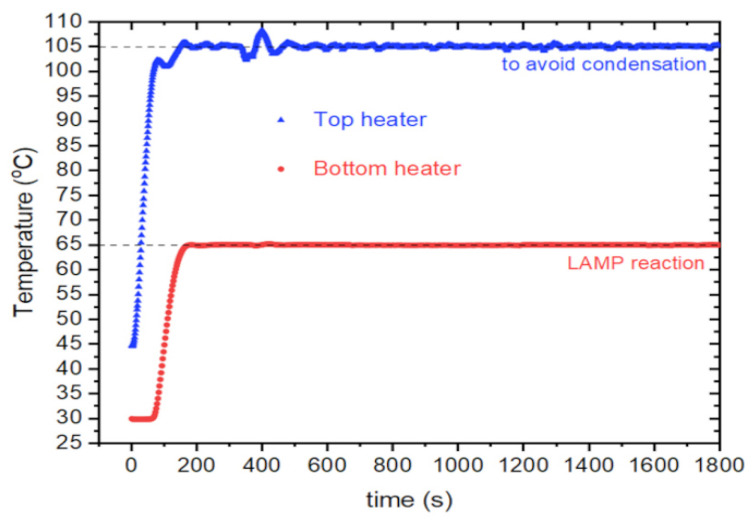
A graph representS the instrument PID control of the temperature, maintaining the top and bottom heaters at the setpoint temperatures during a 30 min time window.

The PID control was implemented using an Arduino program. The PID was crucial for achieving precise temperature control, as highlighted by de Oliveira et al. in 2021 [21]. Their research showcases various low-cost PCR devices utilizing PIDS for accurate temperature control. As illustrated in Figure 6, a representative 30 min cycle exemplifies the PID-controlled conditions, with the bottom set at 65 °C and the top at 105 °C.

The above graph is very similar to the ones observed in commercial instruments, as described by Span et al. in 2019 [32]. The PID parameters were selected based on the system response via manual tuning, wherein the proportional (P) parameter was initially chosen, followed by the integral (I) parameter, and lastly, the derivative (D). This method was successfully employed and implemented by de Oliveira et al. in 2021 [21] on a low-cost PCR system utilizing PCBs as heaters. In the initial phase, the I and D parameters are set to 0, and the P parameter is chosen to obtain the system’s output close to a given setpoint. Once the P parameter is established, the I parameter is adjusted to correct any offset that the output can have in relation to the setpoint, and finally, the D parameter is tuned to increase overall stability in the output. The instrument achieves a temperature precision of ±0.5 °C. It is noted that this difference in variance, when compared to commercial instruments, is expected due to the inherent simplicity of the presented electronics and mechanics.

### 3.2. Colorimetric Detection and Sample Analysis

The assays to test system functionality were carried out following the previously described protocol to ensure its functionality. The tests were performed for the detection of SARS-CoV-2 samples. The instrument is able to analyze eight samples per cycle, with two dedicated positions for positive and negative controls and the remaining six designated for clinical samples. To ensure a comprehensive evaluation, identical conditions and reactions were replicated in an Applied Biosystems ProFlex PCR System thermal cycler using the LAMP configuration, allowing for a comparative analysis of results (see Table 1). In the case of conventional RT-LAMP reactions, result interpretation was conducted visually, relying on direct observation.

The interpretation of the color change must be carefully analyzed. Following the method published in the article by Aoki et al. (2021) [30], reaction outcomes are categorized into pink for negative, yellow for positive, and either orange or an indistinguishable color for cases considered undetermined. The authors categorized undetermined cases to avoid false positives and potential treatment errors. Therefore, for these cases, the standard PCR analysis is strongly recommended.

To evaluate the proposed equipment, we prepared 66 clinical samples previously tested in RT-qPCR, 29 of which were positive and 37 negative.

**Figure 7:**
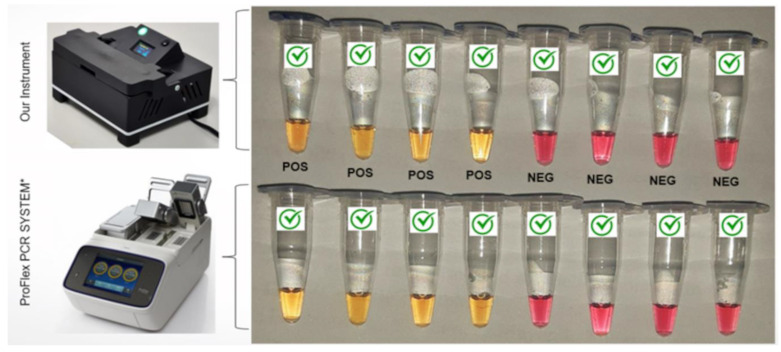
Comparison of RT-LAMP colorimetric reactions performed in a conventional Applied Biosystems ProFlex PCR System thermocycler (used in the LAMP mode) and our instrument. Yellow reactions are considered positive, and pink reactions are negative.

These samples were tested on the equipment proposed in this work and the PROFlex PCR System, configured for the LAMP method (Figure 7). As a result, in PROFlex, we obtained 29 positives and 37 negatives. In the proposed equipment, we obtained 30 positives, 31 negatives, and 5 indeterminates.

As shown in Table 1, our instrument successfully reproduced the conditions to perform the colorimetric reactions in 30 min. The results indicate a concordance of 90.9% when compared to the Applied Biosystems ProFlex PCR System. These data are also in accordance with other instruments reported in the literature. Rodriguez-Manzano et al. (2021) [33], for example, developed a handheld POC device capable of detecting SARS-CoV-2 in under 20 min. The system, however, requires a Bluetooth connection to a smartphone for results interpretation. Ganguli et al., 2020, [34] developed a portable detection system for SARS-CoV-2 capable of distinguishing positive from negative samples within 30 min; however, it relies on a smartphone-based reader for result analysis.

**Figure 8:**
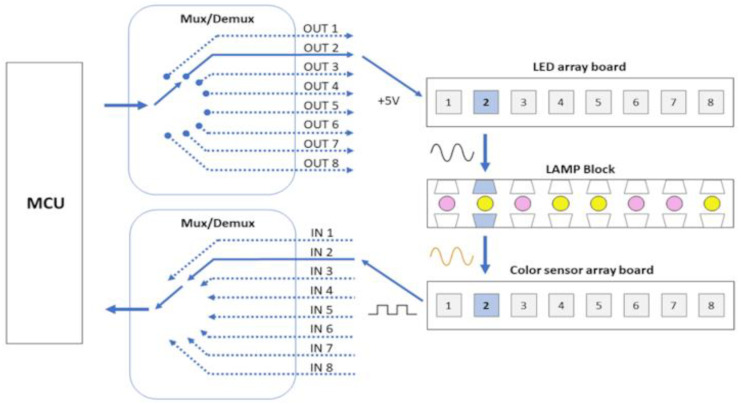
Mux/Demux configuration allows a unique selection of an LED/color sensor pair working per target sample read and reduces the number of MCU pins used.

The results are automatically interpreted and displayed on the OLED screen. This is facilitated by customized color interpretation software. The pH indicator phenol red undergoes a color change in the reaction, transitioning from pink to yellow when the SARS-CoV-2 sample is positive, as can be observed in Figure 6 and Figure 8.

Following the testing and automated interpretation of all 66 clinical samples, sensitivity and specificity were calculated, excluding indeterminate samples. This exclusion was based on the recommendation that indeterminate samples be analyzed using the standard PCR method, as they do not provide specific results. The results obtained indicated a sensitivity of 100% and a specificity of 97.3%.

The role of electronics in the automatic colorimetric detection process is to ensure that only an LED and its respective TCS3200 color sensor work synchronously and also to stabilize the signal through capacitive filters. It is important that only one sensor is read at a time because if we turn on more than one LED or sensor at a time, the light from adjacent samples will influence the reading of the target sample, creating signal noise sources. The Mux/Demux device allows, in addition to saving Arduino processing and pins, a unique selection of an LED/color sensor pair working per target sample read (Figure 8). Despite the hierarchical structure of the reading process, it only takes a few seconds. Additionally, the current passing through the LED, and consequently its intensity, is controlled through resistors. This control ensures that the LED intensity is not excessively high, preventing saturation of the color sensor with white light. Maintaining a low LED intensity is essential to establishing an optimal environment for effective color detection.

**Figure 9:**
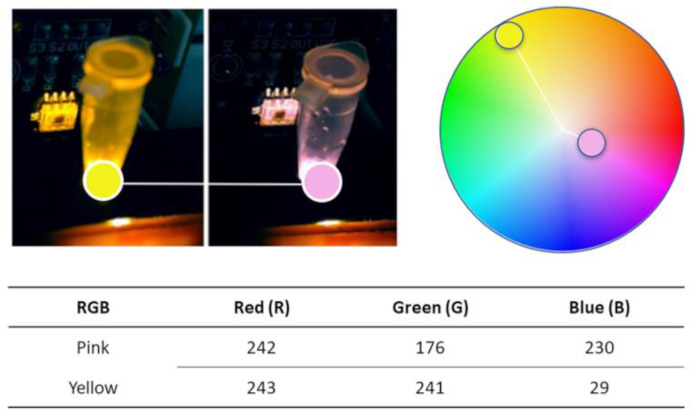
Instrument color detection system demonstration: One board emits a white light via LED; the light travels through the microtubule and carries the color information to the color sensor.

The TCS3200 color sensor has the capability to read three fundamental pieces of information inherent in color: the quantity of red (R), green (G), and blue (B). To determine the critical information to extract from our RT-LAMP results, an Adobe tool capable of extracting the levels of R, G, and B present in an image was employed (https://color.adobe.com/; accessed on 20 March 2022). This approach allows the interpretation of information from positive and negative samples on an RGB scale (0–255). The illustration of this process and the instrument’s colorimetric detection system is shown in Figure 9.

As demonstrated above in Figure 9, the amount of red present negative (pink) and positive (yellow) samples is almost equal, so we do not need to read R. The color wheel, Figure 9, gives us a spatial perspective of the color arrangement and the existing distance between pink and yellow. Knowing this is important for implementing the colorimetric detection firmware, as it saves Arduino nano microcontroller processing power and time. The amount of G and B is the only one with a significant variation. Despite the B color being the one that varies the most, its information alone would not be enough to guarantee the stability and quality of the reading. Using both B and G in the instrument’s color interpretation process ensures that possible reading noise, such as the influence of external light, is minimized. This POC instrument color detection system reads just the G and B frequencies from the samples. The color interpretation begins at the end of the RT-LAMP amplification process after the samples are cooled down to 30 °C to prevent the light from being blocked by the sample condensation on the vial body.

The TCS3200 does not read color in standard RGB format with a range from 0 to 255; instead, it does a light-to-frequency conversion. Even though the value read is not RGB, the variation in green and blue colors still takes place, and a process of calibrating the instrument with real samples makes it capable of interpreting color changes. Approximately 60 clinical samples processed in the commercial Applied Biosystems ProFlex PCR System thermal cycler were used to calibrate our colorimetric detection system. Firstly, the green color frequency (Gf) is read, followed by the blue color frequency (Bf).

We performed a subtraction operation (Gf-Bf) to determine whether the sample is positive, negative, or indeterminate. It is important to note that this color interpretation process by difference does not identify the specific color, which is not a concern for the instrument’s intended application. The interpretation values (Gf-Bf) obtained through calibration are detailed in Table 2. Various methods and technologies exist for SARS-CoV-2 detection in the literature. Other POC instruments have been developed using molecular biology techniques other than PCR and colorimetric RT-LAMP. The complexity of a device directly correlates with its development and production costs on a large scale. Our aim was to develop an instrument capable of replicating the necessary conditions for RT-LAMP reactions, irrespective of the pathogen or the sample’s prior processing, while also providing automatic result interpretation. We have successfully developed an instrument that can be easily and rapidly manufactured on a large scale, utilizing commercial sensors, displays, buttons, and peripheral materials. This addresses the urgent demands of a pandemic, making it suitable for application in remote or rural areas. The instrument only requires electrical energy for operation and is adaptable for use with conventional batteries. This allows it to be configured for independent battery use, and the result interpretation is simple and easy, not requiring trained personnel.

## 4. Conclusions

A low-cost RT-LAMP eight-vial sample instrument was developed employing commercial LEDs, sensors, a 3D-printed housing, and an Arduino Nano board. The instrument features an automatic colorimetric detection system and precise control of sample temperature. Customized PCBs serve as heating elements for temperature adjustment. An aluminum block, aligning the vials and enhancing heating contact, is incorporated, and two small computer fans are utilized for reaction cooling. A PID control system ensures accurate temperature control. A Mux/Demux system, alongside an eight LED array and an eight TCS3200 color sensor array, was used to reduce processing power, optimize resource use, and improve our color detection methodology. Our low-cost RT-LAMP instrument has demonstrated performance comparable to the widely employed commercial equipment, the Applied Biosystems ProFlex PCR System, commonly utilized in laboratories, making it an effective, portable, and user-friendly alternative. Operable on an external 12 V, 5 A power supply, it is adaptable for conventional batteries, aligning with the POC concept for independent battery use. While the design emphasizes the capability to process eight samples, the underlying principles can be scaled to accommodate varying sample quantities. With a total cost of just USD 100, our instrument has successfully undergone testing for SARS-CoV-2 RNA detection. These results affirm the potential of our technology as a viable and cost-efficient alternative for diagnostic applications. The results demonstrate that this technology can serve as a valuable alternative for diagnosis in laboratories with limited financial resources. The initial point to highlight is that LAMP reactions typically do not take place in conventional thermal blocks or water baths. Given the critical need for temperature stability and precision in LAMP reactions, practitioners often resort to using basic PCR instruments without detection systems or opt for quantitative PCR instruments. Consequently, there is a scarcity of commercialized LAMP instruments, with existing ones often requiring proprietary LAMP reaction kits. Notably, standard PCR instruments lack color detection capabilities, relying solely on fluorescence. Consequently, when conducting LAMP on a PCR instrument, the equipment cannot provide an answer; instead, the detection relies on manual visual inspection by the operator. Therefore, our article, showcasing an open-source, portable instrument with color detection capabilities, stands out as a truly unique contribution to the field.

Although the equipment’s viability was demonstrated specifically using SARS-CoV-2 RNA samples, its versatility extends to detecting other viruses and pathogens by employing alternative primers. Additionally, our findings highlight that custom PCBs can function as a heating element not only in lab-on-a-chip devices but also in thermocycler instruments.

## Figures and Tables

**Table 1:** Comparison of RT-LAMP and the Applied Biosystems ProFlex PCR System thermal cycler results in 66 clinical samples.

	Biosystems ProFlex	RT-LAMP Arduino Instrument
Positive	43.9% (*n* = 29)	45.5% (*n* = 30) (1 false positive)
Negative	56.1% (*n* = 37)	83.8% (*n* = 31)
Indeterminate	-	9.1% (*n* = 5)
Total	*n* = 66	*n* = 66

**Table 2:** Color detection system G-B interpretation value range.

Result	(Gf-Bf)
Positive	−20
Negative	25
Indeterminate	−20 < (Gf - Bf) < 25

## Data Availability

Data are contained within the article and Supplementary Materials.

## Supplementary Materials

The following supporting information can be downloaded at: https://www.mdpi.com/article/10.3390/bios14030128/s1, Table S1: Set of RT-LAMP primers originally designed by Rabe and Cepko (2020) to detect SARS-CoV-2 by RT-LAMP and presently used by our research group.
