# Supplementary material for: Low-Cost Arduino Reverse Transcriptase Loop-Mediated Isothermal Amplification (RT-LAMP) for Sensitive Nucleic Acid Detection
Source: Biosensors (Basel). 2024 Feb 29;14(3):128. doi: 10.3390/bios14030128 (PMC10968452; doi:10.3390/bios14030128)
Supplement: Supplementary file 1 [file biosensors-14-00128-s001.zip › biosensors-2852526-supplementary.pdf]

Low-Cost Arduino Reverse Transcriptase Loop-Mediated Isothermal Amplification (RT-LAMP) for Sensitive Nucleic Acid Detection

Bruno Dias Camargo [1,2], Mateus Cassaboni Stracke [1,2], Heloisa Bruna Soligo Sanchuki [1], Viviane Klassen de Oliveira [2], Hellen Cristina Ancelmo [1,2], Dayanne Mozaner Bordin [3,*], Fabricio Klerynton Marchini [1,2], Emilson Ribeiro Viana [4] and Lucas Blanes [1,2]

[1] Laboratory for Applied Science and Technology in Health, Carlos Chagas Institute, Oswaldo Cruz Foundation (Fiocruz), Professor Algacyr Munhoz Mader 3775 St., Curitiba 81350-010, Brazil; bruno.camargo@ibmp.org.br (B.D.C.); mateus.stracke@ibmp.org.br (M.C.S.); heloisa.sanchuki@ibmp.org.br (H.B.S.S.); hellen.ancelmo@ibmp.org.br (H.C.A.); fabricio.marchini@ibmp.org.br (F.K.M.); lucas.blanes@fiocruz.br (L.B.)

[2] Paraná Institute of Molecular Biology, Professor Algacyr Munhoz Mader 3775 St., Curitiba 81350-010, Brazil; klassen.viviane@gmail.com

[3] School of Mathematical and Physical Sciences, Faculty of Science, University of Technology Sydney, Ultimo, NSW 2007, Australia

[4] Academic Department of Physics (DAFIS), Federal University of Technology—Paraná (UTFPR), Sete de Setembro 3165 Av., Curitiba 80230-901, Brazil; emilsonjunior@utfpr.edu.br

* Correspondence: dayanne.bordin@uts.edu.au

Supplementary Table S1. Set of RT-LAMP primers originally designed by Rabe and Cepko (2020) to detect SARS-CoV-2 by RT-LAMP and presently used by our research group.

Primers	Sequence (5’ - 3’)
F3	CGGTGGACAAATTGTCAC
B3	CTTCTCTGGATTTAACACACTT
LF	TTACAAGCTTAAAGAATGTCTGAACACT
LB	TTGAATTTAGGTGAAACATTTGTCACG
FIP	TCAGCACACAAAGCCAAAAATTTATTTTTCTGTGCAAAGGAAATTAAGGAG
BIP	TATTGGTGGAGCTAAACTTAAAGCCTTTTCTGTACAATCCCTTTGAGTG
